# Prevalence and clinical characteristics of uveitic glaucoma: multicentric study in Bogotá, Colombia

**DOI:** 10.1038/s41433-023-02757-9

**Published:** 2023-10-03

**Authors:** William Rojas-Carabali, Germán Mejía-Salgado, Carlos Cifuentes-González, Daniela Chacón-Zambrano, Danna Lesley Cruz-Reyes, Maria Fernanda Delgado, Héctor Fernando Gómez- Goyeneche, Katrina Saad-Brahim, Alejandra de-la-Torre

**Affiliations:** 1 Neuroscience (NEUROS) Research Group, Neurovitae Research Center, Institute of Translational Medicine (IMT), Universidad Del Rosario Escuela de Medicina y Ciencias de la Salud, Bogotá, Colombia; 2 Ophthalmology Interest Group, Neuroscience (NEUROS) Research Group, Neurovitae Research Center, Institute of Translational Medicine (IMT), Universidad Del Rosario Escuela de Medicina y Ciencias de la Salud, Bogotá, Colombia; 3 Grupo de Investigación Clínica. Escuela de Medicina y Ciencias de la Salud, Universidad del Rosario, Bogotá, Colombia; 4 Private Practice and Sociedad de Cirugía Ocular, Bogotá, Colombia; 5 Glaucoma specialist, Diagnóstico Ocular del Country, Bogotá, Colombia

**Keywords:** Uveal diseases, Glaucoma, Ocular hypertension

## Abstract

**Objectives:**

To describe the clinical features of patients diagnosed with uveitic glaucoma (UG) and ocular hypertension secondary to uveitis (OHT-SU).

**Methods:**

A multicentric cross-sectional study using medical records of patients with uveitis between 2013 and 2021. Uveitis and glaucoma specialists examined all patients. Variables were analyzed using the chi-square or Fisher’s exact test for categorical variables. Additionally, *t* test, Mann–Whitney, and Kruskal–Wallis variance analysis were used for continuous variables. Finally, a Kaplan–Meier survival analysis for UG and OHT-SU development over time was done.

**Results:**

Of the 660 clinical records reviewed of patients with uveitis, 191 (28.9%) had OHT-SU in at least one visit, and 108 (16.4%) of them developed UG. In all ages, females were more affected than males. Anterior uveitis was the main anatomic localisation, and non-granulomatous, recurrent, and inactive uveitis were the most frequent clinical features. The mean final visual acuity was 0.3 (0.0–1.0) LogMAR. Also, 95.8% of the patients had additional sequelae related to uveitis regardless of UG and OHT-SU. Interestingly, males had earlier affection, with statistical significance in OHT for adults (*P* = 0.036) and UG for children (*P* = 0.04). Of all patients, 81.1% received topical hypotensive treatment and 29.8% required a surgical procedure.

**Conclusions:**

UG and OHT-SU are common complications of uveitis in the Colombian population. These sight-threatening conditions were more common and appeared sooner in men at any age. Our results suggest that earlier and more aggressive treatment with topical hypotensive agents could positively influence the visual outcomes and the requirement of surgical procedures.

## Introduction

Uveitic glaucoma (UG) is considered one of the most vision-threatening complications in patients with uveitis [1, 2]. Its presentation and frequency depend on age, sex, race, and aetiology of uveitis [3, 4]. Its prevalence in patients with chronic uveitis is around 20%; however, due to its heterogeneous definition, its accurate distribution is not known with precision [5–7]. Additionally, its incidence is directly proportional to the time of evolution of uveitis. Neri et al. described that UG is closely related to the progression time of uveitis, where its incidence progressed from 6.5%, 11.1%, and 22.7% at one, five, and ten years, respectively [8].

Age is a significant risk factor for UG development, as the risk increases proportionally with age, with an OR for patients between 21 to 40 and 61 to 80 years of 9.4 and 30, respectively [9]. As for paediatric patients, its frequency is lower, but it could be more aggressive, generating worse visual outcomes and more bilateral involvement [1, 10].

The aetiologies of uveitis most strongly associated with the development of UG are Herpes virus, Juvenile Idiopathic Arthritis, Fuchs’ heterochromic iridocyclitis, Posner-Schlossman syndrome, and Vogt–Koyanagi–Harada syndrome [3]. Currently, few reports describe the characteristics of patients with UG in Latin America. In Colombia, studies in paediatric population have reported a frequency between 3.3% and 5.2% [11, 12]. Likewise, in the adult population, UG represents the fourth cause of secondary glaucoma [13]; despite this, the clinical characteristics of UG in our population are still poorly described. Therefore, this study aims to describe the clinical characteristics of patients diagnosed with UG and ocular hypertension secondary to uveitis (OHT-SU) in four ophthalmological centres in Bogotá, Colombia, between 2013 and 2021.

## Methods

### Design

Observational cross-sectional study.

### Population

We reviewed 660 clinical records of patients with uveitis who attended four ophthalmology centres in Bogotá, Colombia, between 2013 and 2021. Inclusion criteria were (1) patients diagnosed with UG or OHT-SU, according to the Standardisation of Uveitis Nomenclature (SUN) recommendations [14, 15]. Exclusion criteria were (1) medical records of patients without uveitis confirmed diagnosis, (2) patients who had glaucoma before uveitis, and (3) patients with other optic neuropathy different from glaucoma. After applying the selection criteria, we recovered 191 clinical records of patients with UG and OHT-SU.

### Definitions

OHT-SU was defined as an increase in IOP greater than 21 mmHg, requiring the prescription of at least one treatment for elevated IOP, without evidence of glaucomatous damage, either structural (such as cup/disc ratio or changes observed on Optical Coherence Tomography) or functional (visual field loss). If such damage occurred, the patient was reclassified as having UG.

### Patient approach

Each patient underwent an examination by a uveitis specialist and a glaucoma specialist, each with more than 20 years of experience. The patient was referred to other specialists to determine any suspected underlying systemic or infectious disease if necessary. Follow-ups for the patients were independently conducted by each specialist, tailored to the standard of care based on factors such as the grade of inflammation, progression of glaucomatous damage, the patient’s response to treatment, and prognosis. The interval between patient visits was variable, according to these individual circumstances and care needs. Patients received detailed eye examinations, physical examinations, and paraclinical work-up to diagnose infectious and non-infectious aetiologies, following international guidelines [16]. Posteriorly, uveitis was classified by anatomical location, onset, course, and duration of the disease according to the SUN [15]. Finally, the patient received adequate management to treat ocular and underlying diseases.

### Data collection

We elaborated and validated a database in Microsoft Excel (Microsoft Corp., Redmond, WA, USA) including the variables of interest, which comprised: sociodemographic information, aetiology, clinical characteristics, treatment modalities, and complications. Uveitis was classified into infectious and non-infectious (NIU). Then NIU was subclassified according to the type of immune-mediated disease-related (autoimmune, autoinflammatory, and mixed [autoimmune/autoinflammatory] based on the classification proposed by McGonagle and McDermott and the El-Shebiny et al.‘s actualisation) [17, 18]. Coauthors trained in ophthalmology, especially in uveitis, filled the dataset. As visual acuities are usually recorded with the Snellen scale in feet, we used the Holladay method [19] to convert them to LogMAR.

### Statistical analysis

In the descriptive analysis, the quantitative variables were reported as mean and standard deviation (SD) or median and interquartile range (IQR) (25th–75th percentile) and categorical variables as relatives and absolutes frequencies and percentages. The chi-square (χ2) test or Fisher’s exact test were used to compare categorical variables. Additionally, we used the *t* test to compare continuous variables with normal distribution and the Mann–Whitney test for variables without normal distribution. Also, Kruskal–Wallis nonparametric variance analysis test was used to investigate differences in continuous variables between more than two groups. Furthermore, we used Spearman correlation to evaluate the association between continuous variables. All the analyses were done using jamovi. (Version 1.6) A *P*-value < 0.05 was considered statistically significant in all cases.

Finally, we performed a Kaplan–Meier survival analysis with a previously fitted Cox model estimated to analyse the time of UG and OHT-SU development. This analysis was done using software R, version 4.0.4. (The R Foundation for Statistical Computing, Vienna, Austria).

## Results

### Uveitis aetiology and characteristics

In the review of 660 clinical records of patients with uveitis, OHT-SU was observed in at least one visit for 191 patients, accounting for 28.9% of cases. Among these patients with OHT-SU, glaucomatous damage was detected and reclassified as UG in 108 cases, representing 16.4% of the total sample. Females were the most affected (F:M ratio 1.34:1), with a median age at first uveitis episode of 49.0 years (IQR 33.0–65.0). Anterior uveitis was the main anatomic localisation of inflammation, and non-granulomatous, recurrent, and inactive uveitis were the most frequent clinical features. Autoinflammatory was the primary type of uveitis (36.11%), and the mean final visual acuity was 0.3 (0.0–1.0) logMAR. In both groups, idiopathic, ocular toxoplasmosis, and herpetic uveitis were the most common aetiologies. UG group had a larger cup-to-disc (C/D) ratio when compared with the OHT-SU group (*P* < 0.001) and required higher dosing and number of hypotensive medications (*P* = 0.003). Additionally, the mean time of progression from OHT-SU to UG was 4.52 years (IC 95% 2.29 years–6.74 years). More detailed information is in Table 1 and Table 2.

**Table 1:** Characteristics of uveitis in patients with UG and OHT-SU.

Clinical characteristics	UG *N* = 108 (%)	OHT-SU *N* = 83 (%)	*P-*value^a^
Sex: Female/Male:	62 (57.40%)/46 (42.59%)	48 (57.83%)/35 (42.16%)	0.953
Ethnicity:			
Latino	106 (98.14%)	83 (100%)	0.51
Asian	1 (0.93%)	-	
European	1 (0.93%)	-	
Bilateral compromise	45 (41,66%)	44 (53.01%)	0.119
Age of first uveitis episode mean (IQR 25th and 75th):	49.0 (33.0– 65.0)	46.0 (31.0–60.0)	0.379
Follow-up time in weeks	169.1 (SD 189.3)	107.4 (SD 156.7)	0.070
Age > 60 years	37 (34.25%)	26 (31.32%)	0.497
Bilateral compromise	45 (41.66%)	44 (53.01%)	0.119
Type of inflammation			
Granulomatous	16 (14.81%)	9 (10.84%)	0.399
Non-granulomatous	88 (81.48%)	72 (86.74%)	
Missing data	4 (3.70%)	2 (2.40%)	
Anatomic Localisation			
Anterior	57 (52.57%)	46 (55.42%)	0.126
Anterior + Intermediate	1 (0.92%)	0 (0%)	
Intermediate	3 (2.77%)	8 (9.63%)	
Posterior	6 (5.55%)	1 (1.20%)	
Panuveitis	38 (35.18%)	27 (32.53%)	
Missing data	3 (2.77%)	1 (1.20%)	
Course:			
Acute	14 (12.96%)	11(13.25%)	0.558
Chronic	40 (37.03%)	36 (43.37%)	
Recurrent	54 (50.0%)	35 (42.16%)	
Missing data	0 (0%)	1 (1.20%)	
Aetiology			
Autoimmune	13 (12.03%)	13 (15.66%)	0.933
Autoinflammatory	39 (36.11%)	28 (33.73%)	
Infectious	20 (18.51%)	17 (20.48%)	
Mixed	8 (7.40%)	6 (7.22%)	
Others	27 (25.0%)	18 (21.6%)	
Missing data	1 (0.92%)	1 (1.20%)	
Worst anterior chamber cellularity:			
0+	35 (32.40%)	23 (27.71%)	0.623
0.5+	14 (12.96%)	16 (19.27%)	
1+	19 (17.59%)	12 (14.45%)	
2+	19 (17.59%)	13 (15.66%)	
3+	13 (12.03%)	12 (14.45%)	
4+	6 (5.55%)	6 (7.22%)	
Missing data	2 (1.85%)	1 (1.20%)	
Worst anterior chamber flare:			
0+	89 (82.40%)	66 (79.51%)	0.664
0.5+	2 (1.85%)	3 (3.61%)	
1+	9 (8.33%)	6 (7.22%)	
2+	0 (0%)	5 (6.02%)	
3+	2 (1.85%)	0 (0%)	
4+	4 (3.70%)	2 (2.40%)	
Missing data	2 (1.85%)	1 (1.20%)	
Worst vitreous cellularity:			
0+	73 (67.59%)	42 (50.60%)	0.074
0.5+	6 (5.55%)	12 (14.45%)	
1+	8 (7.40%)	9 (10.84%)	
2+	10 (9.25%)	6 (7.22%)	
3+	3 (2.77%)	5 (6.02%)	
4+	1 (0.92%)	0 (0%)	
Missing data	7 (6.48%)	9 (10.84%)	
Worst vitreous haze:			
0+	74 (68.51%)	51 (61.44%)	0.658
0.5+	1 (0.92%)	4 (4.81%)	
1+	10 (9.25%)	5 (6.02%)	
2+	9 (8.33%)	9 (10.84%)	
3+	4 (3.70%)	2 (2.40%)	
4+	5 (4.62%)	4 (4.81%)	
Missing data	5 (4.62%)	8 (9.63%)	
Final visual acuity (LogMAR):	0.3 (0.0–1.0)	0.3 (0.0–1.3)	0.380
Hypopyon	2 (1.85%)	1 (1.20%)	0.721
Cataract	68 (62.96)	41 (49.39%)	0.060
Anterior Synechiae	13 (12.03%)	4 (4.81%)	0.082
Posterior Synechiae	39 (36.11%)	20 (24.09%)	0.075
Trabeculitis	8 (7.40%)	5 (6.02%)	0.707
Time topical steroids (weeks)	9 (1.0–33.2)	12.5 (4.0–61.9)	0.186
Mean number of hypotensive agents	3.0 (2.0–3.0)	2.0 (1.0–3.0)	**0.003**
Last Visit Cup/Disc ratio	0.5 (0.3–0.8)	0.2 (0.1–0.3)	**<0.001**
Highest IOP (IQR 25th and 75th) [Range]	28 (18.0–35.7) [10–56]	22.0 (18.0–28.0) [6–62]	0.089

^a^A *t* test or Mann–Whitney test was used for continuous variables, and a Chi-square (χ2) test or Fisher’s exact test for categorical variables according to their distribution. Statistically significant *p*-values are in bold.

**Table 2:** Uveitis etiologies in secondary ocular hypertension and glaucoma.

Aetiology	UG		OHT-SU	
	*N*	%	*N*	%
Idiopathic	26	24.1	18	21.7
Anterior viral uveitis^a^	14	13	9	10.8
Toxoplasmosis	6	5.6	7	8.4
Ankylosing spondylitis	3	2.8	1	1.2
HLA-B27+	3	2.8	2	2.4
Juvenile idiopathic arthritis	3	2.8	3	3.6
Multiple Sclerosis	2	1.9	1	1.2
Post-traumatic Uveitis	2	1.9	1	1.2
Sarcoidosis (Probable or confirmed)	2	1.9	3.6	1.2
Crohn’s disease	1	0.9	0	0.0
Mixed connective tissue disease	1	0.9	1	1.2
Granulomatosis with polyangiitis	1	0.9	0	0.0
Ocular lymphoma	1	0.9	0	0.0
Sympathetic ophthalmia	1	0.9	0	0.0
Psoriasis	1	0.9	0	0.0
Psoriatic arthritis	1	0.9	1	1.2
Rheumatoid arthritis	1	0.9	2	2.4
Sjögren syndrome	1	0.9	2	2.4
Suspect Behçet	1	0.9	0	0.0
Tuberculosis	1	0.9	0	0.0
Ulcerative colitis	1	0.9	0	0.0
Birdshot retinochoroidopathy	0	0.0	1	1.2
Endophthalmitis	0	0.0	1	1.2
Lens-induced uveitis	0	0.0	1	1.2
Peripheral spondylarthritis	0	0.0	1	1.2
Reactive arthritis	0	0.0	2	2.4
Vogt–Koyanagi–Harada syndrome	0	0.0	3	3.6
Undetermined	35	32.4	23	27.7
Total	108	100	83	100

^a^Patients diagnosed with Herpes Simplex Virus, Varicella Zoster Virus, or Cytomegalovirus, confirmed through Polymerase Chain Reaction (PCR) or suspected based on clinical presentation.

Furthermore, in the analysis of complications beyond UG and OHT-SU among the 191 patients studied, 95.8% developed additional sequelae related to uveitis, independent of UG and OHT-SU. An association was observed between the occurrence of relapses and the development of these sequelae (*P* < 0.001). Cataracts (57.1%), keratic precipitates (55.5%), posterior synechiae (30.9%), and macular oedema (21.5%) were the most common uveitis sequelae. Other sequelae included epiretinal membrane (18.8%), retinal detachment (13.6%), anterior synechiae (8.9%), and vasculitis (7.3%). When comparing the complications between patients with UG and OHT-SU, corneal oedema (*P* = 0.017), posterior synechia (*P* = 0.008), and the neovascularization of the anterior segment (*P* = 0.019) were statistically more frequent in patients with UG. For more detailed information on complications, see Supplementary material–T1.

We recorded the highest levels of IOP evidenced during follow-up. These levels positively correlated with anterior chamber cellularity (*r* = 0.227, *P* = 0.003). However, there was no statistical correlation between anterior chamber flare, vitreous cellularity, or vitreous haze with IOP. Additionally, patients with corneal oedema had higher mean IOP of 34.1 mmHg (SD 13.2) vs. 24.8 mmHg (SD 10.3) (*P* = 0.002), same as those with vasculitis 31.6 mmHg (SD 13.3) vs. 25.3 mmHg (SD 10.6) (*P* = 0.042).

### Characteristics of the UG

From the UG group, 86 had open-angle glaucoma, and 22 had closed-angle glaucoma. There were no significant differences in age, sex, aetiology, IOP, corneal oedema, visual field (VF) abnormalities, OCT abnormalities, and medical or surgical management. Table 3 compares the clinical characteristics between open-angle and closed-angle glaucoma. Regarding the pathophysiology of UG, steroid-induced glaucoma was identified as the cause in nine cases. In 14 cases, the primary cause of glaucoma wastrabeculitis associated with viral anterior uveitis. However, a clear cause-and-effect relationship could not be established in several cases. It is plausible that these instances involved a mix of multiple mechanisms.

**Table 3:** Open-angle vs. closed-angle uveitic glaucoma.

Sociodemographic and clinical characteristics	Open-angle glaucoma *N* = 86 (%) Median (IQR 25–75%)	Closed-angle Glaucoma N = 22 (%) Median (IQR 25–75%)	*P-*value^a^
Age of first uveitis episode	48.0 (34.0–64.0)	56.0 (33.0–67.0)	0.520
Female	49 (57.0%)	12 (54.5%)	0.837
Male	37 (43.0%)	10 (43.5%)	
Aetiology	*n*(%)	*n*(%)	0.38
Idiopathic	13 (15%)	5 (23%)	
Toxoplasmosis	5 (5.8%)	1 (4.5%)	
Herpes simplex virus	5 (5.8%)	1 (4.5%)	
Juvenile idiopathic arthritis	3 (3.5%)	0 (0%)	
HLA-B27+	2 (2.3%)	0 (0%)	
Cytomegalovirus	2 (2.3%)	0 (0%)	
Tuberculosis	2 (2.3%)	0 (0%)	
Mixed connective tissue disease	2 (2.3%)	0 (0%)	
Sarcoidosis	1 (1.2%)	1 (4.5%)	
Post-traumatic Uveitis	1 (1.2%)	1 (4.5%)	
Rheumatoid arthritis	1 (1.2%)	1 (4.5%)	
Herpes zoster virus	1 (1.2%)	1 (4.5%)	
Multiple Sclerosis	1 (1.2%)	0 (0%)	
Psoriasis	1 (1.2%)	0 (0%)	
Sjögren syndrome	1 (1.2%)	0 (0%)	
Reactive arthritis	1 (1.2%)	0 (0%)	
Post-traumatic Uveitis	1 (1.2%)	0 (0%)	
Fuchs’ heterochromic iridocyclitis	1 (1.2%)	0 (0%)	
Granulomatosis with polyangiitis	1 (1.2%)	0 (0%)	
Viral unspecific	1 (1.2%)	0 (0%)	
Suspect Behçet	1 (1.2%)	0 (0%)	
Crohn’s disease	0 (0%)	1 (4.5%)	
Ocular lymphoma	0 (0%)	1 (4.5%)	
Undetermined	37 (43%)	6 (27%)	
Highest IOP (IQR 25th and 75th) [Range]	27 (18.0–35.0) [8–56]	28 (15.0–36.0) [6–62]	0.962
Final IOP	15 (14.0–19.3)	16 (13.0– 20.0)	0.838
Last visit Cup/Disk ratio	0.5 (0.35–0.9)	0.7 (0.55–0.8)	0.622
Corneal oedema	12 (14.63%)	3 (3.65%)	0.847
Abnormal VF	38 (%)	8 (%)	0.554
Abnormal OCT	38 (%)	4 (6%)	0.748
Number of hypotensive agents	3.0 (2.0–3.0)	2.5 (1.2–3.0)	0.303
Need for a surgical procedure	43 (%)	8 (%)	0.283

^a^A *t* test or Mann–Whitney test was used for continuous variables and a Chi-square (χ2) test or Fisher’s exact test for categorical variables according to their distribution.

### Comparison between the types of uveitis

Autoimmune uveitis had an earlier first uveitis episode and required surgical procedures in more patients than other aetiologies. Furthermore, infectious uveitis had a higher mean IOP and more frequent abnormal VFs. However, none of these associations had a significant *P*-value in the Kruskal–Wallis analysis. More information is available in Table 4.

**Table 4:** Clinical characteristics and visual outcomes in patients with UG and OHT-SU according to the type of uveitis.

Type of uveitis	*N*	Age at the first episode of uveitis Median (25–75%)	Sex *N* (F/M ratio):	Final BCVA (logMAR) Median (25–75%)^a^	Final C/D ratio Median (25–75%)^a^	Highest IOP Median (IQR 25th and 75th) [Range]^a^	Abnormal Visual field *N* (%)	Abnormal OCT *N* (%)	Mean of hypertensive agents used Median (25–75%)^a^	Surgically Managed *N* (%)
Autoimmune	26	41.0 (24.0–57.0)	(19/7) 2.7:1	0.48 (0.09–2.10)	0.5 (0.3– 0.6)	22.0 (17.5–29.0) [8–50]	6 (23.0%)	7 (26.9%)	2.0 (1.0–3.0)	10 (38.4%)
Autoinflammatory	67	45.0 (28.5–57.3)	(38/29) 1.3:1	0.39 (0.0–1.30)	0.4 (0.2–0.6)	23.0 (18.0–31.0) [6–54]	17 (25.3%)	21 (31.3%)	2.0 (1.0–3.0)	19 (28.3%)
Infectious	37	55.5 (42.0–66.7)	(14/23) 0.6:1	0.20 (0.0–0.61)	0.3 (0.2–0.5)	27.5 (18.2–32.0) [8–62]	12 (32.4%)	10 (27.0%)	3.0 (1.0–3.0)	10 (27.0%)
Mixed	14	48.0 (43.2–55.2)	(8/6) 1.3:1	0.04 (0.0–0.20)	0.5 (0.1–0.8)	20.5 (15.5–27.5) [9–34]	3 (21.4%)	3 (21.4%)	2.0 (0.0–2.7)	2 (14.2%)
Others	47	57.0 (37.0– 67.0)	(29/16) 1.8:1	0.39 (0.09–0.88)	0.4 (0.2–0.9)	25.0 (17.7–33.5) [8–56]	12 (25.5%)	11 (23.4%)	3.0 (2.0–3.0)	16 (34.04%)

*BCVA* Best-corrected visual acuity, *C/D* Cup/Disc, *IOP* intraocular pressure, *OCT* optical coherence tomography.

^a^Kruskal–Wallis nonparametric variance analysis test was used to compare continuous variables between groups.

### A subanalysis of the paediatric population

Of the 191 patients, there were 20 children. UG was present in 10 and OHT-SU in 10. As in adults, females were more affected than males. Panuveitis was the most common uveitis anatomic localisation, followed by anterior and intermediate. This population seems to have more OCT abnormalities than VF abnormalities. More information is available in Supplementary material–T2.

### UG and OHT-SU development during follow-up

**Fig. 1:**
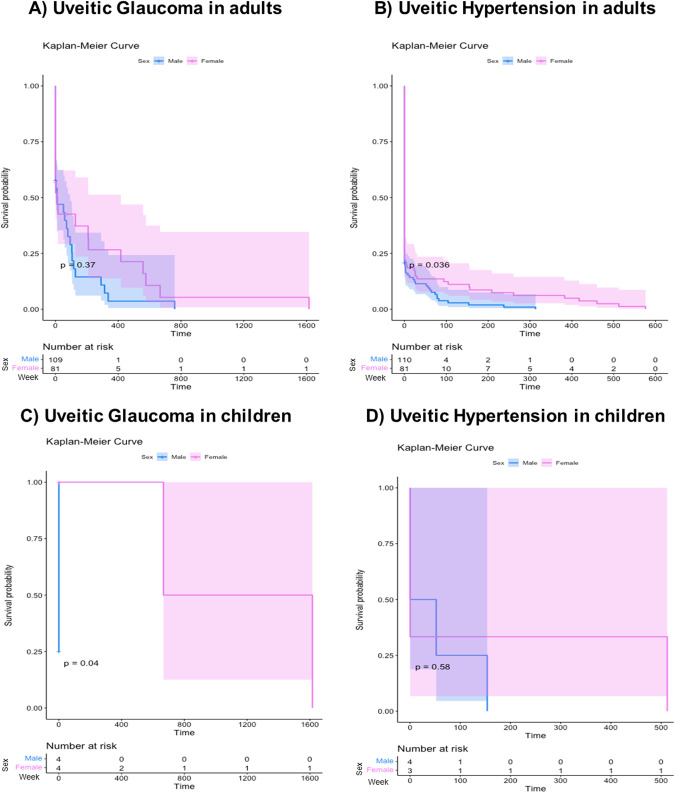
Kaplan–Meier survival analysis for UG and OHT-SU post uveitis diagnosis. **A** Kaplan–Meier survival analysis comparing UG diagnosis between genders in adults found no significant difference in the time of glaucoma onset (*P* = 0.37), even though UG seemed to progress slightly more quickly in men. The one-year survival rate was 70.8% (95% CI 62.3–80.4%) for women and 66.9% (95% CI 56.8–78.9%) for men. This trend persisted into the second year, with survival rates of 61.55% (95% CI 52.21–72.6%) for women and 58.17% (95% CI 47.36–71.4%) for men. Half of men and women were diagnosed with UG within the first three and four years, respectively. **B** Kaplan–Meier survival analysis for OHT-SU revealed a significantly shorter time to diagnosis in men (*P* = 0.036). Half of the men were diagnosed with OHT-SU within 38 weeks, whereas women achieved this milestone after a year (65 weeks). **C** In paediatric onset uveitis, the Kaplan–Meier survival analysis for UG indicated a significantly earlier diagnosis in boys compared to girls. All the boys were diagnosed, in contrast to girls, where only half were diagnosed after approximately four years (230 weeks). In one female patient, UG was diagnosed after a history of 30 years with uveitis. **D** For OHT-SU, the Kaplan–Meier survival analysis did not suggest any significant impact of gender on the timing of diagnosis (*P* = 0.58). However, the progression was observed to be faster in 50% of the cases in women, while men typically took around a year to receive their diagnosis.

Around 75% of adults and 50% of children arrived at the first visit with the uveitis specialist with ocular hypertension and glaucoma. In both groups, males were affected earlier, with statistical significance in OHT for adults (*P* = 0.036) and UG for children (*P* = 0.04). Figure 1 illustrates the UG and OHT-SU development since the first visit with a Kaplan–Meier survival analysis in adults (Fig. 1A, B) and children (Fig. 1C, D). Interestingly, there was not a significant difference in the mean follow-up between both groups (UG vs. OHT) (*P* = 0.07) (Supplementary material–F1)

### UG and OHT-SU treatment

Among the patients diagnosed with UG and OHT-SU, 155 (81.1%) received topical hypotensive therapy. Beta-blockers were the most frequently utilised hypotensive agents, used in 77% of patients, followed by topical carbonic anhydrase inhibitors in 65.4%, alpha2-agonists in 56.5%, oral carbonic anhydrase inhibitors in 17.3%, and prostaglandin analogues in 13.1%. The remaining 36 patients (18.2%) who did not receive topical hypotensives had already undergone surgical interventions by the time they arrived at our centres or they had instances of transient high intraocular pressure. Supplementary material–T3.

Likewise, 57 patients (29.8% of 191 patients with OHT-SU or UG) required a surgical procedure, with a median time between diagnosis and surgical procedure of 24 months (IQR 4–45 months (in 51 patients)). Borderline statistically significant differences were found between UG (median 13 months (IQR 4–40 months [in 43 patients])) and OHT-SU patients (median 36 months (IQR 24–86 months [in 8 patients])) (*P* = 0.058). The Ahmed valve was the glaucoma drainage implant (GDI) used in all cases and was the surgical procedure most frequently performed, followed by peripheral iridotomy and trabeculectomy. Of the patients who underwent the first procedure, 7 (12%) required a second procedure. Among these cases, the first procedure was Ahmed valve implantation in 3 (43%), iridectomy in 2 (29%), selective laser trabeculoplasty in 1 (14%), and trabeculectomy in 1 (14%). More detailed information about treatment can be found in Supplementary material–T3.

## Discussion

Glaucoma is one of the leading causes of moderate to severe visual impairment and irreversible blindness worldwide [20]. The UG can develop as a severe complication of inflammation or due to the prolonged use of systemic or topical corticosteroids [8]. The prevalence data of UG varies according to the definition of glaucoma, age at presentation, and type of uveitis [9]. Several studies use a definition of UG based on elevated IOP alone [8, 9]; however, this concept has evolved throughout the years, and glaucoma definition requires the evidence of structural damage of the optic nerve [15].

The incidence of UG is similar among subtypes of uveitis [8]; however, some authors have reported that anterior uveitis has a higher incidence of UG and elevated IOP [21]. Herbert et al. reported a prevalence of OHT-SU of 46.1% in eyes with chronic uveitis compared to 26% in eyes with acute uveitis [7], and a recent study reported the prevalence of UG in 4.1%, of which 92.4% had open-angle glaucoma [22]. Furthermore, Kanda et al. [23] reported a higher frequency of UG in patients with chronic granulomatous ocular inflammation (64.5%) than in acute non-granulomatous inflammation (35%). Similarly, our results showed a higher proportion of UG in chronic and recurrent than in acute uveitis. This could be due to the trabecular meshwork’s morphological changes that occur over time, secondary to long-lasting inflammation and the prolonged use of corticosteroids [23]. In the same way, Neri et al. reported an incidence of glaucoma of 7.6% in acute uveitis and 11.1% in chronic uveitis at five years [8]. We found a prevalence of 16.4% of UG after approximately 3.5 years (169.1 weeks), with a high frequency of bilateral involvement (41.66%).

Notably, around 75% of adults and 50% of children arrived at the first visit with the uveitis specialist with ocular hypertension and glaucoma. This, in addition to the frequency of bilateral compromise, leads to a high risk of poor visual outcomes and demonstrates that the referral process to uveitis specialists in our country is complex due to the scarcity of specialist and administrative procedures. Therefore, healthcare professionals and general ophthalmologists must be aware of this disease to diagnose it early and refer to the uveitis specialist with adequate laboratory tests. [24]

Tekeli et al. [22], in a cross-sectional study, reported an anterior compromise of 72.4% in a group of 105 eyes with UG followed by panuveitis (18.6%), intermediate uveitis (6.2%), and posterior uveitis (2.7%), similar to the results of Sharon et al. [25] in a long-term clinical study between 2003 and 2015 in 53 eyes with UG, where 83% of the eyes had anterior uveitis. In our results, anterior uveitis was also the most common site of inflammation in both the UG group (52.57%) and the OHT-SU group (55.42%) (*P* = 0.126), followed by panuveitis. Hence, inflammation in the anterior chamber may play an important role, or it could correspond with the frequency of occurrence, considering that the anterior is the most common anatomical localisation of uveitis [5, 8].

Moreover, we found a positive correlation between the grade of anterior chamber inflammation and the IOP levels that aligns with the more significant proportion of UG and OHT-SU in patients with anterior uveitis [25]. This could be explained because the high aqueous flare and cellularity in those patients produce more precipitation of proteins, inflammatory cells, and debris in the trabecular meshwork, generating a drop in the aqueous humour outflow. Evidence that inflammatory cells lead to higher resistance of the trabecular outflow derives from studies that recorded acute rises in IOP after Nd: YAG laser capsulotomy [26, 27]. Nevertheless, some uveitis with acutely raised IOP, such as Posner-Schlossman syndrome, have low levels of inflammation activity in the anterior chamber [28], implying that other mechanisms like trabeculitis contribute to the increased IOP, as in the case of herpetic uveitis [29].

Additionally, older age increases the risk of elevated IOP [7]. We found that the median age of presentation was in the fourth decade of life. Extremes of age are associated with higher susceptibility to corticosteroid induce OHT; the highest risk has been reported in children of 4 to 6 years old and elderly patients [30]. Nevertheless, Sijssens et al. reported elevated IOP development in 35% of patients under 16 with uveitis [10]. Additionally, in Fuchs’ Heterochromic Iridocyclitis and Posner-Schlossman Syndrome, classically hypertensive entities, the median age of onset is 20–60 years, with no gender predilection [30].

For this multi-centre-based study, idiopathic was the primary aetiology, followed by *Herpes Simplex Virus*, Toxoplasmosis, and seronegative spondyloarthropathies-associated uveitis. Following our results, previous studies report that UG can be a complication of idiopathic uveitis [8], herpetic uveitis [31], and juvenile idiopathic arthritis-associated uveitis. [10] However, we had a higher proportion of infectious aetiologies, which may be related to Colombia being a developing country where infectious diseases, especially *Toxoplasma gondii* infection, are more prevalent than in developed countries. [32, 33]

Our study found that the time between diagnosis and surgical procedures was similar to the reported by Carreño E et al. [34] In their study, most patients took more than a year to undergo a surgical procedure, similar to our median of 13 months in patients with UG. However, our study showed a lower frequency of patients requiring reintervention compared to that reported by Carreño E et al. [34] This difference might be attributed to the different surgical approaches that were mainly used in each side, being trabeculectomy in the previous study and Ahmed valve implantation in our patients.

Comparing clinical features of patients with OHT-SU who developed or did not develop glaucoma, nerve damage (measured by C/D ratio) was more considerable in the UG group (median 0.5) than in the OHT-SU group (median 0.2) (*P* < 0.001). This correlates with the criteria applied to define UG, where there must be structural damage to the optic nerve by clinical measurement of C/D or paraclinical with abnormal OCT featuring a decrease in nerve fibre layer thickness or functional loss evidenced in the VF. Likewise, it correlates with the natural history of glaucomatous damage since cupping and OCT abnormalities increase over time according to the number, duration, and intensity of ocular hypertension episodes. [35, 36]

Tekeli et al. found differences in visual acuity and optic disc characteristics in Turkey. They described that infectious uveitis had visual acuities between 0.4 and 0.7 and non-infectious uveitis between 0.5 and 0.9 LogMAR, worse than those found in our population in both cases. Similarly, they reported C/D ratios and IOP much worse than our patients [22]. This can be due to differences in the follow-up time between both studies and the aggressiveness of treatment. On average, we used more hypotensive medications, which can directly influence these clinical results. Additionally, we found better visual outcomes and final C/D ratios in infectious aetiologies than non-infectious, maybe because inflammatory control could be slower in NIU.

On the other hand, VF defects were also more frequent in the patients described by Sharon et al. where most patients had non-infectious aetiologies; 49% developed a VF defect, which is higher than our population [25]. The type of patients may influence this difference since Sharon et al. reported only patients with UG, unlike ours, where patients with OHT were also included.

Our findings related to the choice of hypotensive agents align with those reported by Merayo-Lloves et al. [5], and they are also consistent with the guidelines and protocols suggested for patients with UG [4, 37]. Despite the proven efficacy of prostaglandin analogues in reducing IOP in uveitis patients and their low probability of inducing complications such as anterior inflammation, cystoid macular oedema, and herpes simplex reactivation [38, 39], our results reflect real-world practice. In these settings, clinicians, aware of the potential complications, tend to favour other categories of hypotensive agents.

Additionally, in terms of uveitis management, treating the underlying cause for prompt and sustained control of inflammation over time is crucial and is indirectly associated with changes in the IOP. [30] Although our study did not aim to outline the treatment pattern of uveitis per se, we emphasise the critical role of systemic medication, including the use of corticosteroid-sparing immunomodulatory therapy, in impacting visual health. [30]

Regarding the requirement for surgical treatment, it has been described that about 30% of patients with UG undergo surgical treatment [4, 37]. Our findings align with this, as 29.8% of the 191 patients with OHT-SU in our study required surgical intervention due to inadequate IOP control despite receiving triple topical and oral hypotensive therapy. Both trabeculectomy and GDI procedures have demonstrated positive outcomes in reducing IOP. In our study, GDI was the most commonly performed procedure, reflecting surgeon preferences and providing evidence that GDI is an effective treatment strategy with higher cumulative success and a longer duration of effectiveness. [40]

Kothari et al. analysed paediatric patients with UG and NIU, finding that females were predominantly affected [41]. Another study in which the highest proportion of aetiologies was non-infectious also showed a 6:1 f:m ratio [25]. Our results coincide, as we found a F:M ratio between 1.3:1 and 2.7:1 in non-infectious aetiologies. These results are expected considering that females have a higher risk of presenting immune-mediated diseases than men [42]. In contrast, the f:m ratio was 0.6:1 for the infectious aetiologies, which is in line with the published by Toniolo et al., who found that in a cohort of patients with UG due to Fuchs’ heterochromic iridocyclitis, 60% of their patients were males. These data and ours suggest that infectious aetiologies could have some relationship with the development of UG in males [43]. More prospective studies are needed to prove this possible association.

On the other hand, previous studies have described the male gender as a risk factor for developing UG and OHT-SU [44, 45]. However, others reported that the need for glaucoma surgery was significantly associated with the female gender [46]. Interestingly, we found that men, young and adults, developed UG and OHT-SU earlier than women. This could reflect a biological susceptibility of men or be explained by the trend of delayed health help-seeking when they become ill, influenced by sociocultural and behavioural factors [47, 48]. Either way, this reinforces gender-dependent differences in access to health and visual outcomes, so more emphasis is required on this topic.

This study presents limitations, notably its retrospective design, variability in follow-up periods, and extended intervals between patient visits. Despite patients receiving specialist care, a significant number still developed UG. This could potentially be attributed to the extended waiting times for appointments with uveitis or glaucoma specialists. Notably, in Colombia, the number of ophthalmologists, and particularly subspecialists, is limited. [24] In addition, we cannot ensure medication adherence, especially in patients with extended intervals between visits. Another limitation encountered in this study was the inability to distinguish the specific pathophysiology of UG in several cases, potentially due to mixed mechanisms involved in the pathophysiology. This factor could influence the interpretation of outcomes and the selection of treatment approaches for these particular cases. Despite these limitations, this study offers important insights into the practical circumstances in developing countries where access to healthcare presents more significant challenges. Additional research is required to assess the impact of glaucoma, specifically UG, on vision loss due to missed examinations and delayed follow-up appointments.

## Conclusion

UG and OHT-SU are common complications of uveitis in the Colombian population, affecting a higher percentage of patients with uveitis than those reported in other parts of the world. These sight-threatening conditions appear earlier in men of all ages and are related more commonly to anterior, non-granulomatous, unilateral, and chronic or recurrent uveitis. Our findings suggest that male patients have a higher probability of progressing to UG before the first consultation and a greater likelihood of progressing to ocular hypertension than women. Consequently, rigorous follow-up in male patients could enhance treatment outcomes.

## Summary

### What was known before:

- Uveitic glaucoma is a common complication of chronic uveitis.
- Uveitic glaucoma is a potentially blinding disease.

### What this study adds:

- For the first time, we reported the clinical characteristics of a large cohort of patients with uveitic glaucoma in a Latin American country.

### Supplementary information

Supplementary Material 1

## Data Availability

The corresponding author can share the information in the databases used in this article upon reasonable request.
