# Supplementary material for: Prevalence and clinical characteristics of uveitic glaucoma: multicentric study in Bogotá, Colombia
Source: Eye (Lond). 2023 Oct 3;38(4):714–22. doi: 10.1038/s41433-023-02757-9 (PMC10920824; doi:10.1038/s41433-023-02757-9)
Supplement: Supplementary file 1 — Supplementary Material 1 [file 41433_2023_2757_MOESM1_ESM.pdf]

T1. Complications of uveitis in patients with UG and OHT-SU

Characteristic	N	UG = 108 (57%)	OHT-SU= 83 (43%)	*P*-value[a]
**Corneal edema**	191	17 (16%)	4 (4.8%)	**0.017**
**Posterior vitreous detachment**	191	38 (35%)	27 (33%)	0.7
**Macular edema**	191	22 (20%)	19 (23%)	0.67
**Epiretinal membrane**	191	22 (20%)	12 (14%)	0.29
**Keratic precipitates**	191	57 (53%)	46 (55%)	0.72
**Band keratopathy**	191	6 (5.6%)	4 (4.8%)	>0.99
**Retinal detachment**	191	13 (12%)	12 (14%)	0.62
**Vitreous hemorrhage**	191	5 (4.6%)	2 (2.4%)	0.7
**Bullous keratopathy**	191	3 (2.8%)	1 (1.2%)	0.63
**Anterior synechia**	191	13 (12%)	4 (4.8%)	0.082
**Posterior synechia**	191	43 (40%)	18 (22%)	**0.008**
**Retinal neovascularization**	191	3 (2.8%)	2 (2.4%)	>0.99
**Periphlebitis**	191	1 (0.9%)	1 (1.2%)	>0.99
**Retinoschisis**	191	2 (1.9%)	0 (0%)	0.51
**Ciliary rotation**	191	0 (0%)	2 (2.4%)	0.19
**Neovascularization of the anterior segment**	191	7 (6.5%)	0 (0%)	**0.019**
**Iridian nodules**	191	5 (4.6%)	5 (6.0%)	0.75

[a] A t-test or Mann–Whitney test was used for continuous variables, and a Chi-square ($\chi 2$) test or Fisher's exact test for categorical variables according to their distribution. Bold= Statistically significant.

T2. Pediatric uveitic glaucoma and secondary ocular hypertension

Clinical characteristics	UG<br>N=10 (%)	OHT-SU<br>N=10 (%)
Female<br>Male	7 (70%)<br>3 (30%)	6 (60%)<br>4 (40%)
**Age of first uveitis episode:**	7.5 (5.0 - 12.0)	5.0 (3.5 - 10.7)
**Anatomic Localization**<br>Anterior<br>Intermediate<br>Panuveitis	 3 (30%)<br>1 (10%)<br>6 (60%)	 4 (40%)<br>2 (20%)<br>4 (40%)
**Etiology**<br>Autoimmune<br>Autoinflammatory<br>Idiopathic<br>Infectious<br>Mixed<br>Others	 3 (30%<br>0 (0%)<br>4 (40%)<br>0 (0%)<br>0 (0%)<br>3 (30%)	 2 (20%)<br>1 (10%)<br>5 (50%)<br>1 (10%)<br>1 (10%)<br>0 (0%)
**Last visit visual acuity (logMAR):**	0.3 (0.2- 0.4)	0.0 (0.0 - 0.1)
**Highest IOP**	22 (15.0 - 31.5)	29.0 (21.2 - 32.2)
**Last visit Cup/Disk ratio**	0.7 (0.5 - 0.7)	0.1 (0.0 - 0.1)
**Abnormal OCT**	5 (50%)	4 (40%)
**Abnormal VF**	3 (30%)	0 (0%)
**# Hypotensive medications**	2.5 (1.2 - 3.7)	1.5 (0.0 - 2.0)
**Surgically managed**	7 (70%)	0 (0%)

T3. Medical and surgical management used in UG and OHT-SU

Medical treatment, n= 155 (81.15%)		
**Hypotensive**	**UG**	**OHT-SU**
Beta-blockers	85	62
Topical carbonic anhydrase inhibitor	76	49
Alpha2-agonist	69	39
Oral carbonic anhydrase inhibitor	24	9
Prostaglandin analogs	20	5
**Surgical treatment, n= 57 (29.84%)**		
**Surgical procedure**	**UG**	**OHT-SU**
Ahmed valve implant	22	0
Trabeculectomy	13	1
Peripheral iridotomy	13	4
Surgical Iridectomy	6	1
SLT	3	1

F1. Mean follow-up time for the UG and OHT-SU groups.

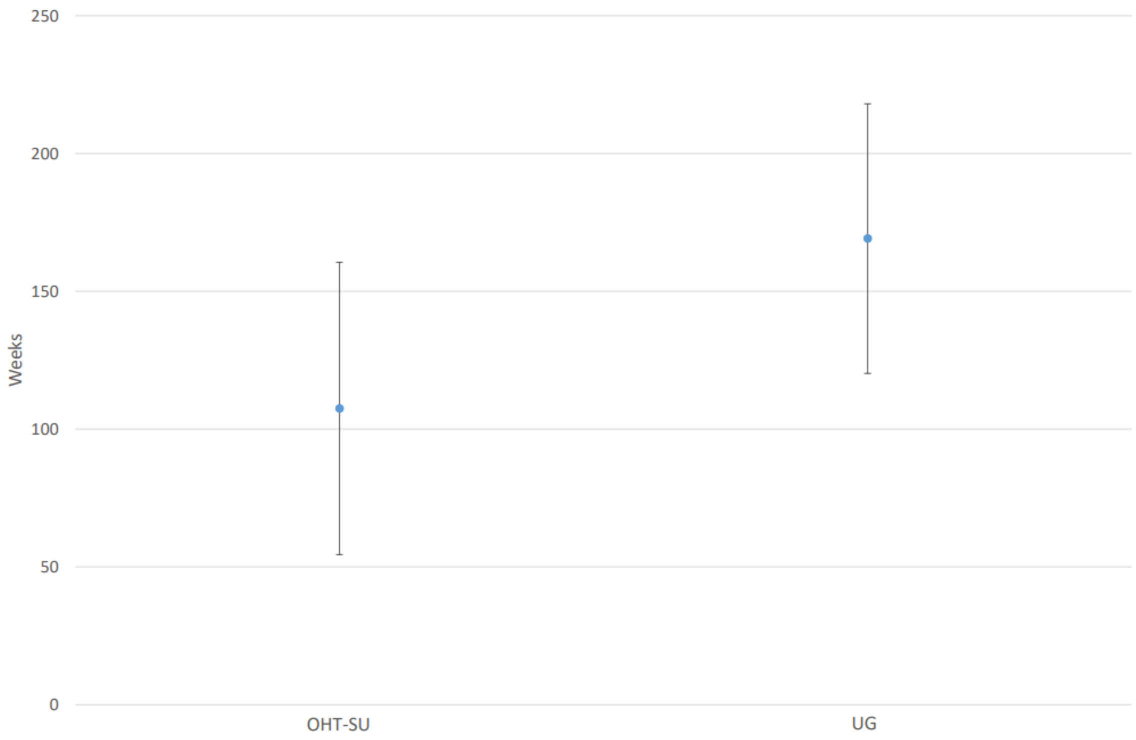

The mean follow-up time was 107.4 weeks in the OHT-SU group vs. 169.1 weeks in the UG group.

P= 0,07 (T-test)
